# Investigating the Multiple Regulatory Mechanisms and Therapeutic Targets of PHLDA1 in Neurological Diseases

**DOI:** 10.2174/011570159X413561251125064110

**Published:** 2026-01-13

**Authors:** Xiaodong Liu, Zhengxiang Lv, Gaoyuan Xu, Yu Chen, Haijun Liu, Ping Xu

**Affiliations:** 1 Department of Neurology, Affiliated Hospital of Zunyi Medical University, Zunyi, China

**Keywords:** PHLDA1, apoptosis, immunoinflammatory response, oxidative stress, pyroptosis, neurological diseases

## Abstract

PHLDA1 (pleckstrin homology-like domain family A member 1) is a pleiotropic regulatory protein that affects key biological processes such as apoptosis, pyroptosis, immune inflammation, autophagy, metabolism, and oxidative stress. PHLDA1 plays a significant role in the pathological mechanisms of neurological diseases. This article systematically reviews the molecular characteristics of PHLDA1 and its core role in cerebrovascular diseases such as cerebral ischemia/reperfusion injury, cerebral hemorrhage, subarachnoid hemorrhage, epilepsy, amyotrophic lateral sclerosis (ALS), and Parkinson's disease (PD). Studies have shown that PHLDA1 promotes disease progression by regulating signalling pathways such as the NF-κB, MAPK, NLRP3 inflammasome, PPARγ, and Nrf2 pathways, thereby exacerbating neuroinflammation, mitochondrial dysfunction, endoplasmic reticulum stress, and pyroptosis in neurons. Its expression is regulated by the dynamic balance of miRNAs (such as miR-194 and miR-101), transcription factors (Egr1 and BHLHE40), and heat shock proteins (HSPs/HSF1). In addition, PHLDA1 has become a potential target for intervention in neurodegenerative and ischemic injuries by inhibiting FundC1-mediated mitochondrial autophagy, regulating microglial polarization, and activating TRAF6-dependent neuroinflammation. This article not only clarifies the pathogenic mechanism of PHLDA1 but also summarizes the relevant intervention strategies targeting PHLDA1, hoping to provide a corresponding theoretical basis and reference for the development of precision therapies for neurological diseases.

## 1 INTRODUCTION

PHLDA1, also known as TDAG51, PHRIP, and DT1P1B1, was originally identified as a proapoptotic protein. Early studies have demonstrated its ability to bind to T-cell receptors and induce Fas expression, thereby triggering apoptosis [1]. Additionally, PHLDA1 functions as a p53 regulatory protein that is located primarily in the plasma membrane of cells, where it participates in cell signal transduction processes. Its expression has also been detected in the perikaryon, nucleus, and nucleolus [2], indicating its close association with the regulation of cell proliferation, apoptosis, and other signalling pathways. There are two protein subtypes of PHLDA1: the long subtype, which contains 401 amino acid residues, and the short subtype, which consists of only 260 amino acid residues. Notably, the short subtype is expressed predominantly in various cancer cells [3]. PHLDA1 is expressed at reduced levels in various cancers, including melanoma, breast cancer, oral cancer, and gastric adenocarcinoma, leading to its classification as a tumor suppressor [4, 5]. As research has progressed, PHLDA1 has been found to play a significant role in the pathogenesis of diseases affecting various systems, including the respiratory, circulatory, digestive, urinary, nervous, and other systems. In the nervous system, PHLDA1 contributes to disease pathogenesis through its diverse biological functions and complex regulation of various signalling pathways. This paper provides a detailed introduction to the biological characteristics of PHLDA1 and the biological functions of the associated signalling pathways, with a particular emphasis on the specific role of PHLDA1 in signalling pathways related to nervous system diseases. A comprehensive understanding of these topics is crucial for the advancement of diagnostic and treatment strategies for nervous system disorders.

### 1.1 Study Design

This review employs systematic literature retrieval and analysis methods. By searching databases such as PubMed and Web of Science, a combination of keywords covering PHLDA1 (and its aliases), core signalling pathways (such as NF-κB, NLRP3, PPARγ, and Nrf2), and target neurological diseases (cerebral ischemia/reperfusion injury, cerebral hemorrhage, epilepsy, ALS, and Parkinson's disease) were used to collect relevant experimental studies (cell and animal models) and clinical association studies. Strict inclusion criteria were established, and two researchers independently conducted title/abstract screening, as well as full-text rescreening. The extracted data were structurally classified and analyzed according to the molecular characteristics of PHLDA1, regulatory pathways (apoptosis, pyroptosis, inflammation, autophagy, metabolism, and oxidative stress), and disease types. The key approach is to adopt a strategy of thematic induction and signalling pathway integration to analyze the core mechanism of PHLDA1 within the multidimensional pathological network and to systematically present its role as a "molecular hub" and targeted intervention strategies through schematic diagrams and tables. In the analysis, research evidence with clear mechanisms and causal verification (such as genetic manipulation) should be prioritized to ensure the reliability of the conclusion to the greatest extent possible. This review involves narrative integration, and no meta-analysis has been conducted.

## 2 THE BIOLOGY OF PHLDA1 AND ITS FAMILY MEMBERS

The PHLDA gene family consists of three members: PHLDA1, PHLDA2, and PHLDA3 [6]. Their distribution within the organization varies, and their different domain combinations endow each member with unique functions.

PHLDA2 is an imprinted gene localized to 11p15.5 and is expressed primarily in the placenta. It plays a critical role in signal transduction among various trophoblastic cells, coordinating the growth of the trophoblastic layer and maintaining placental stability, as well as supporting embryo growth and development by influencing cyclin and apoptosis-related proteins [7]. An increase in placental PHLDA2 expression leads to the upregulation of apoptosis-related gene expression, which subsequently enhances the apoptosis of trophoblastic cells and diminishes their proliferative capacity. This cascade results in placental insufficiency, potentially impacting fetal development and weight gain in mild cases, whereas in severe cases, it can cause ischemia and hypoxia and induce systemic arteriolar spasm in pregnant women, which is closely associated with the onset of preeclampsia [8, 9].

The PHLDA3 gene is located at the 1q32.1 position and contains a p53-binding region, making it a direct target gene of the p53 protein. Like PHLDA1, PHLDA3 is capable of inducing apoptosis, with its function closely linked to the PH domain. Overexpression of PHLDA3 impairs the development of vasoblasts and intersegmental vascular cells. Studies have shown that the PH domain of PHLDA3 is homologous to that of AKT and performs similar molecular functions, competing with AKT for binding to diphosphorylphosphatidylinositol (3,4-PIP2) and triphosphorylphosphatidylinositol (3,4,5-PIP3). This competition inhibits the migration and activation of AKT to cell membranes, thereby participating in the regulation of apoptosis, migration, proliferation, and other biological processes [10]. In pancreatic neuroendocrine tumors, lung neuroendocrine tumors, and rectal neuroendocrine tumors, PHLDA3 has been shown to lose function due to loss of heterozygosity and methylation [11-13].

The PHLDA1 gene, located on chromosome 12q15, encodes two subtypes: the long subtype, which has 401 amino acid residues, and the short subtype, which has 260 amino acid residues. Although the mechanisms of action and target objects of PHLDA1 are similar to those of PHLDA2 and PHLDA3, the PH domain structure of PHLDA1 differs from those of the other two. PHLDA1 contains a split-like PH domain, which, while composed of seven β-folds and one α-helix, is divided into N-terminal (β-folds 1-3) and C-terminal (β-folds 4-7 and the α-helix) segments, separated by more than 40 amino acid residues containing polyglutamine (QQ). A fully functional PH domain is formed only when both ends are combined [6]. The PH domain is capable of binding phosphatidylinositol phosphate, thereby localizing the host protein to the cell membrane, facilitating interactions with other membrane proteins, providing a signalling complex or cytoskeletal scaffold, and folding the catalytic domain within the same protein to prevent it from accessing its substrate.

Additionally, as a scaffold protein, it enhances the signalling efficiency of upstream and downstream effectors, promoting their synergistic interactions [14]. Proline-rich proteins typically involve multiple protein associations, such as with vesicle-associated proteins, whereas glutamate-rich proteins are involved in transcriptional regulation and neurogenesis [15]. PHLDA1 can regulate various biological processes through different factors, including proliferation, apoptosis, migration, and invasion, and plays a crucial role in the occurrence and development of multiple mechanisms, such as oxidative stress and the immune response.

PHLDA1 is highly expressed in the lungs and pancreas of mammals, with moderate expression observed in the brain, heart, placenta, liver, and kidneys. Various factors and cytokines can alter the expression level of PHLDA1, thereby participating in diverse pathophysiological processes [16, 17]. In conclusion, the diversity of the biological functions of PHLDA1 arises from the complexity of its biological characteristics.

## 3 PHLDA1-RELATED SIGNALLING PATHWAYS

### 3.1 PHLDA1 is Associated with Apoptosis-Related Signalling Pathways

Apoptosis, the principal form of programmed cell death, is essential for maintaining tissue homeostasis and eliminating damaged cells [18]. Among the various apoptotic pathways, the mitochondrial apoptotic pathway is a pivotal mechanism characterized by alterations in mitochondrial outer membrane permeability (MOMP), the release of cytochrome c (Cyt c), and the activation of the apoptotic protease cascade [19]. PHLDA1 serves as a critical regulatory hub in this pathway. Studies indicate that PHLDA1 directly influences the balance of Bcl-2 family proteins during MOMP. Specifically, PHLDA1 plays a dual role akin to that of BH3-only proteins: on the one hand, it binds to the proapoptotic protein Bax (targeting its N-terminal or hydrophobic channel), facilitating the translocation of Bax from the cytoplasm to the mitochondrial membrane and promoting its oligomerization; on the other hand, it disrupts the inhibitory effects of anti-apoptotic proteins (such as Bcl-2 and Mcl-1) on Bak at the mitochondrial membrane, thereby releasing and activating Bak for oligomerization. The resulting activated Bax/Bak oligomers ultimately induce MOMP, leading to the release of proapoptotic factors, such as cytochrome c and Smac/ DIABLO, and irreversible initiation of the apoptotic program [20]. Furthermore, PHLDA1 is implicated in the regulation of the death receptor pathway, which is activated by extracellular death signals (such as TNF-α and FasL) *via* their respective receptors (such as TNFR1 and Fas). Current evidence suggests that PHLDA1 may represent a crucial node linking extracellular signals to the execution of apoptosis. For example, upon activation of the PKC signalling pathway, PHLDA1 can be upregulated, thereby increasing the expression of Fas death receptors and significantly enhancing cellular sensitivity to death signals, such as Fas, thereby amplifying apoptotic signals [21].

As a key transcription factor, p53 activates apoptotic programs in response to DNA damage and other stress signals. Studies have demonstrated that PHLDA1 can bind to p53 and affect its transcriptional activity, thereby regulating p53-dependent apoptotic pathways [2, 22]. Additionally, p53 can bind to the promoter region of the PHLDA1 gene and directly regulate its expression by recruiting P300 and CBP, which alters the acetylation and methylation levels of the promoter region [23]. Peroxisome proliferator-activated receptor γ (PPARγ) is a nuclear factor that regulates the transcription of numerous downstream genes, including those related to apoptosis [24]. The mitochondrial apoptosis pathway is the most well-studied pathway of apoptosis, and PHLDA1 may regulate mitochondria-related processes through PPARγ, thereby facilitating apoptosis [25]. The molecular chaperone STUB1 possesses E3 ligase activity, which is involved in regulating energy metabolism pathways and plays a crucial role in the degradation of abnormal proteins [26]. STUB1 acts as the E3 ligase for PHLDA1. Under harmful stimuli, PHLDA1 misfolds, and STUB1 promotes its ubiquitination and degradation, thereby increasing apoptosis [27].

PHLDA1 plays a significant role in tumorigenesis and development, influencing the fate of tumor cells by regulating the Fas signalling pathway. The Fas receptor, known for its ability to induce apoptosis in tumor cells upon ligand binding, plays a critical role in this process. Studies in hepatocellular carcinoma (HCC) have demonstrated that the expression levels of miR-3682-3p and miR-155-3p are significantly elevated. These two microRNAs directly target and inhibit the expression of PHLDA1. Given that PHLDA1 promotes Fas expression, its inhibition by miR-3682-3p and miR-155-3p leads to the downregulation of Fas expression, thereby attenuating the apoptotic signal mediated by Fas. This mechanism manifests as inhibited proliferation of HCC cells and enhanced apoptosis [28, 29]. This discovery elucidates the crucial role of the PHLDA1-Fas pathway in the occurrence and development of HCC, identifying a potential molecular target for liver cancer treatment. In gastric cancer, the absence of PHLDA1 expression significantly promotes tumor progression. Research has indicated that PHLDA1 effectively inhibits the proliferative capacity of gastric cancer and undifferentiated adenocarcinoma cells. Notably, miR-101 significantly reduces PHLDA1 mRNA expression in gastric cancer cells through direct targeting and binding [30]. Conversely, in osteosarcoma, PHLDA1 has a cancer-promoting effect. It not only enhances the proliferation of osteosarcoma cells but also induces apoptosis while inhibiting the migration, invasion, and colony formation of tumor cells. Further studies suggested that miR-526b-5p positively regulates this process by targeting and modulating PHLDA1 expression [31]. These findings suggest that PHLDA1 may be involved in tumorigenesis and development through distinct molecular mechanisms in various types of tumors and that its specific function depends on the cellular origin and microenvironmental characteristics of the tumor.

In summary, PHLDA1 influences apoptosis through various signalling pathways and plays a critical regulatory role in balancing cell survival and death.

### 3.2 PHLDA1 and Immunoinflammatory Signalling Pathways

The immune-inflammatory response serves as the fundamental mechanism through which the body defends itself against pathogens, injuries, and stress. Its precise regulation is contingent upon a complex signalling network. As illustrated in Fig. (**1**), PHLDA1, a key inflammatory regulatory factor, is intricately integrated into this network and plays a particularly significant role in regulating the core transcription factor NF-κB [32]. The activation of NF-κB represents a common hub activated by various proinflammatory signals, including TLRs, TNF-α, and IL-1β, which drive the expression of numerous inflammatory factors. Notably, PHLDA1 regulates NF-κB through a unique "enhanced" mechanism: studies indicate that PHLDA1 may inhibit IKK activity by interacting with the IKK complex, a crucial kinase for NF-κB activation. This effect may be contradictory; however, it is a clever mechanism that accelerates the phosphorylation and degradation of the NF-κB inhibitor IκBα. The rapid clearance of IκBα releases NF-κB from its cytoplasmic anchor, significantly promoting its nuclear translocation and transcriptional activity [33]. These findings suggest that PHLDA1 circumvents the classical IKK activation pathway and directly "unlocks" NF-κB by hastening the clearance of IκBα, thereby enabling a more effective response to upstream stimuli. Furthermore, the influence of PHLDA1 extends to the downstream effects of NF-κB, as it has been confirmed to further regulate the expression levels of key inflammatory factors, such as IL-6 and TNF-α [34, 35], thereby forming a positive feedback loop. This dual regulation of core transcription factor activity and downstream target gene expression positions PHLDA1 as a potent amplifier of inflammatory signals, profoundly influencing the intensity and persistence of inflammatory responses.

The MAPK signalling pathway is a crucial immunoinflammatory signalling pathway comprising three major MAPK family members: ERK, JNK, and p38 [36]. These kinases play crucial roles in cellular stress responses, inflammation, and immune reactions. Studies have demonstrated that TLR2 stimulation can upregulate the expression of PHLDA1, a process that relies on the activation of the JAK2-ERK1/2-STAT3 signalling pathway. Specifically, TLR2 initiates signal transduction through JAK2, with ERK1/2 serving as an intermediary that mediates the activation of STAT3, thereby promoting the expression of PHLDA1. This pathway is crucial in the immune-inflammatory response [17]. Moreover, PHLDA1 is implicated in the onset and progression of sepsis-induced acute kidney injury (AKI) through its regulation of the JNK/p38 and ERK signalling pathways. Inhibition of PHLDA1 has been shown to significantly mitigate the inflammatory response and oxidative stress injuries associated with AKI [37].

Toll-like receptors (TLRs) play pivotal roles in recognizing pathogen-associated molecular patterns and injury-associated molecular patterns, which induce inflammatory responses through the activation of the NF-κB and MAPK signalling pathways. Recent studies have indicated that PHLDA1 plays a vital role in TLR-mediated immunoinflammation and that the knockdown of PHLDA1 can diminish macrophage activation [17, 38].

The NLRP3 inflammasome is a crucial component of the innate immune system and primarily mediates pyroptosis and the inflammatory response. The activation of the inflammasome is typically triggered by various stimuli, such as crystals, toxins, and oxidative stress, which leads to the activation of Caspase-1 and the subsequent production of inflammatory cytokines [39]. In a subarachnoid hemorrhage model, the downregulation of PHLDA1 has been proven to inhibit the NLRP3 inflammasome signal, inhibit the proinflammatory phenotype of microglia, and promote the activation of the anti-inflammatory phenotype, thereby alleviating SAH-induced brain injury [40]. As a transcription factor, FoxO1 has diverse functions. It binds to the transcriptional promoters of inflammatory factors within the cell nucleus, thereby promoting their transcription.

Additionally, FoxO1 regulates inflammatory responses by modulating the TLR4 pathway [41]. Studies have demonstrated that PHLDA1, a novel regulatory factor of the transcription factor FoxO1, binds to the region between FoxO1 residues 1 and 38. This binding competitively inhibits the interaction of the 14-3-3 protein with FoxO1, thereby promoting its nuclear accumulation and facilitating lipopolysaccharide-induced inflammatory responses [34]. Furthermore, PHLDA1 directly interacts with TRAF6, enhancing its K63-linked polyubiquitination. Ubiquitinated TRAF6 functions as a scaffold protein, recruiting and activating the TAK1 (TGF-β-activated kinase 1) complex while also facilitating the assembly of the IKK (IκB kinase) complex. This process stimulates the NF-κB pathway, which induces inflammation [35]. In models of liver ischemia-reperfusion injury, studies have shown that miR-194 exerts a protective effect by targeting and inhibiting the expression of PHLDA1. An increase in PHLDA1 expression antagonizes the protective effect of miR-194, activating the MAPK and IKK signalling pathways, which promote inflammation and exacerbate liver injury [42]. This regulatory axis underscores the pivotal role of PHLDA1 in liver ischemia-reperfusion injury, suggesting that targeting the miR-194/PHLDA1/MAPK-IKK pathway may represent a novel therapeutic approach.

### 3.3 PHLDA1 and Pyroptosis-Related Signalling Pathways

Pyroptosis is a form of programmed cell death primarily mediated by inflammasomes and is often closely associated with the inflammatory response. In recent years, an increasing number of studies have focused on PHLDA1 and the pyroptosis-related signalling pathways it participates in, as shown in Fig. (**2**). PHLDA1 may influence the initiation and execution of pyroptosis by modulating the expression or activity of various components of these inflammasomes.

PHLDA1 plays a crucial role in the physiological regulation of cells. Studies have demonstrated that PHLDA1 can inhibit Akt activity [6, 16]. Akt is pivotal in regulating pyroptosis by promoting the phosphorylation of FOXO1, which subsequently downregulates the expression of pyroptosis-related proteins, including NLRP3 and IL-1β, while inhibiting the activation of GSDMD. These molecular events are interconnected and ultimately reduce the extent of pyroptosis and oxidative damage to cells, significantly increasing cell survival and viability [43, 44]. During the pathological process of acute lung injury (ALI) induced by sepsis, NLRP3 is activated, leading to the hydrolytic activation of IL-1β and IL-18, which is mediated by caspase 1, ultimately inducing pyroptosis. Relevant studies have demonstrated that silencing PHLDA1 can downregulate the expression of NLRP3, primarily because PHLDA1 reduces the assembly of key components of inflammasomes, such as ASC and procaspase-1, and inhibits the activation of caspase-1, thereby suppressing pyroptosis in cells [45]. As a transcription factor, Nrf2 can inhibit NLRP3-mediated inflammatory responses and apoptosis [46]. However, overexpression of PHLDA1 can promote the expression of Nrf2 nuclear export protein (CRM1), accelerating the transport of Nrf2 from the nucleus to the cytoplasm and inhibiting the associated signalling pathways of Nrf2. Rac1 plays a critical role in activating the NLRP3 inflammasome by regulating the generation of reactive oxygen species (ROS) [47]. Specifically, Rac1 promotes ROS production by activating NADPH oxidases, such as NOX2. As the core signalling molecules for NLRP3 inflammasome activation, ROS can induce the assembly of the inflammasome, including the oligomerization of NLRP3, ASC, and procaspase-1, leading to its subsequent activation. Ultimately, this process results in the maturation and release of IL-1β and IL-18, which are dependent on caspase-1, and pyroptosis of cells [48]. This signalling pathway is highly important in various inflammatory responses, including infections and metabolic diseases.

The elevated expression of PHLDA1 leads to a direct interaction with TRAF6, thus amplifying the K63-linked ubiquitination-mediated activation of NF-κB signalling. Studies have shown that in rats treated with MPTP, PHLDA1 directly binds to TRAF6 [35]. In addition, overexpression of PHLDA1 leads to an increase in Rac1 ubiquitination, whereas overexpression of TRAF6 exacerbates the increase in Rac1 ubiquitination induced by PHLDA1. Shu *et al*. reported that PHLDA1 can enhance sevoflurane-induced pyroptosis in rat neonatal cells through this pathway [49]. PHLDA1 systematically and powerfully promotes pyroptosis through four mechanisms: inhibiting the protective pathway (Akt), directly assembling the pyroptosis executive complex (NLRP3 inflammasome), releasing antioxidant defence (Nrf2 nuclear output), and activating upstream trigger signals (TRAF6-Rac1-ROS). This multitarget and synergistic regulatory model highlights the role of PHLDA1 in the inflammatory cell death network and explains its key pathogenic role in various inflammatory diseases. Targeting PHLDA1 or its downstream effector molecules is expected to become a new strategy for preventing hyperpyroptosis and related diseases.

### 3.4 PHLDA1 is Associated with Metabolic Signalling Pathways

PHLDA1 plays a significant regulatory role in metabolic processes. Elevated expression of PHLDA1 was observed in the podocytes and glomeruli of diabetic mice following exposure to high glucose. PHLDA1 inhibits the phosphorylation of Akt, thereby reducing its activity. This decrease in Akt activity results in dephosphorylation of its downstream target, GSK-3β, which subsequently enhances GSK-3β activity. Activated GSK-3β phosphorylates Nrf2, promoting its recognition and degradation by E3 ubiquitin ligases, thus inhibiting the antioxidant function of Nrf2. Inhibition of PHLDA1 can alleviate the suppression of Nrf2, thereby protecting against podocyte damage induced by high glucose [50].

PPARs, a class of nuclear receptors, serve as major transcriptional regulators of lipid production [51]. Most domains of PHLDA1 bind to specific domains of PPARγ, preventing RXRα from being recruited to PPARγ to form the RXRα heterodimer complex during lipogenesis, thereby inhibiting fat synthesis [52]. The SREBP family is a class of transcription factors that regulate the expression of genes involved in fat synthesis, including those encoding fatty acid synthase and acetyl-CoA carboxylase [53]. PHLDA1 may inhibit the activation of fat synthesis genes by obstructing the nuclear translocation and cleavage of SREBP, thus suppressing fat synthesis. Additionally, PHLDA1 may influence the lipid synthesis capacity of adipocytes by regulating the expression of SREBP [54, 55]. FGF21 is a metabolic regulator that promotes fatty acid oxidation and autophagy while inhibiting fat synthesis by activating its receptors FGFR1 and β-Klotho [56]. PHLDA1 may enhance the promotion of fatty acid oxidation by increasing the expression of FGF21, thereby promoting lipolysis, and may also influence fat metabolism by regulating the activity of downstream molecules in the FGF21 signalling pathway [5, 57].

TGF-β1 is a major profibrotic cytokine, and studies have indicated that PHLDA1 may serve as a regulatory factor that promotes the translation of TGF-β1. TGF-β1 can induce collagen production, leading to an increased demand for protein folding and the splicing phosphorylation of IRE1 and XBP1, thereby promoting fibrosis [58]. A deeper understanding of the role of PHLDA1 in fat metabolism is crucial for elucidating the molecular basis of fat metabolism regulation and for developing treatments for related diseases.

PHLDA1 serves as a double-edged sword in metabolic regulation. It significantly inhibits fat synthesis by disrupting the PPARγ/RXR complex and targeting SREBP while also promoting fat breakdown through FGF21 signalling. However, under pathological conditions, such as diabetic nephropathy, PHLDA1 tends to act more as a 'destroyer', exacerbating oxidative damage by inhibiting the Akt/Nrf2 axis and potentially triggering profibrotic signals *via* the upregulation of TGF-β1. This multitarget and multieffect regulatory model underscores the pivotal role of PHLDA1 in metabolism. A comprehensive analysis of its mechanisms of action is essential not only for elucidating the regulatory networks involved in lipid metabolism, oxidative stress, and fibrosis but also for providing compelling scientific evidence for the development of novel targeted therapies for metabolic diseases, including diabetic complications, fatty liver disease, and fibrotic diseases.

### 3.5 PHLDA1 is also Involved in Oxidative Stress-Related Signalling Pathways

Oxidative stress refers to a condition in which there is an imbalance between the production and clearance of reactive oxygen species within cells, resulting in impaired cellular function [59]. This phenomenon is closely associated with the onset and progression of various diseases, including cardiovascular diseases, neurodegenerative disorders, and cancer [60-62]. Nrf2 is a key regulator of the cellular protective response, activating the expression of various antioxidant genes by binding to antioxidant response elements, such as NQO1, HO-1, and GCLC [63]. Research has demonstrated that PHLDA1 may regulate the degradation of Nrf2, prolonging its duration of activity within cells and further enhancing the expression of antioxidant genes.

Nrf2 is a crucial regulator of neuronal apoptosis, oxidative stress, and the inflammatory response in hippocampal cells subjected to oxygen-glucose deprivation and reoxygenation in mice. The inhibition of PHLDA1 promotes the nuclear translocation of Nrf2 and enhances its transcriptional activity by regulating the phosphorylation of GSK-3β, thereby exerting a neuroprotective effect [64]. Furthermore, PHLDA1 contributes to ischemic and hypoxic-induced brain injury in newborns by inhibiting FundC1-mediated phagocytosis of neurons [65]. JNK, a member of the MAPK family, plays a significant role in oxidative stress and apoptosis. The activation of JNK leads to the phosphorylation of c-Jun, which enhances its transcriptional activity and subsequently affects apoptosis and the inflammatory response [66]. PHLDA1 may mitigate the activation of downstream target genes by inhibiting JNK phosphorylation, thereby alleviating cell damage caused by oxidative stress [37]. In cardiomyocytes, during myocardial ischemia-reperfusion injury induced by oxidative stress, PHLDA1 is highly expressed and interacts with Bax, with this interaction being enhanced under H_2_O_2_ stimulation. The overexpression of PHLDA1 in cardiomyocytes can exacerbate oxidative stress-induced myocardial injury, whereas PHLDA1 knockdown can ameliorate the myocardial cell damage and ischemia-reperfusion injury induced by oxidative stress [20]. One study highlighted the role of PHLDA1 in the oxidative stress-induced apoptosis of mouse embryonic fibroblasts (MEFs), indicating that oxidative stress significantly increases PHLDA1 expression. In contrast, intracellular reactive oxygen species production is markedly elevated in PHLDA1-deficient MEFs, leading to caspase-3 activation. These findings suggest that PHLDA1 plays a protective role in oxidative stress-induced cell death in MEFs [34].

In summary, a variety of signalling factors and pathways can directly or indirectly influence the expression of the PHLDA1 protein. Moreover, the signalling molecules associated with PHLDA1 expression and their functions vary across different diseases, types of stimulation or injury, and cell types.

We summarize the mechanisms through which PHLDA1 regulates the oxidative stress network under various pathological conditions, including Nrf2 stability, mitochondrial quality control, and the integration of apoptotic signals. We aim to provide insights that may aid in the development of targeted intervention strategies focused on this molecule for combating oxidative stress-related diseases, such as cardiovascular diseases and neurodegenerative disorders.

## 4 PHLDA1 AND NEUROLOGICAL DISEASES

PHLDA1 is implicated in the pathogenesis of neurological diseases through various signalling pathways, as illustrated in Fig. (**3**). Each of these pathways contributes to distinct pathological processes, including mitochondrial autophagy, neuroinflammation, apoptosis, pyroptosis, and oxidative stress, ultimately resulting in neuronal damage or even cell death.

### 4.1 PHLDA1 and Cerebrovascular Diseases

In recent years, the association between PHLDA1 and cerebrovascular diseases has gradually attracted attention. As shown in Fig. (**4**), PHLDA1 participates in the pathogenesis of cerebrovascular diseases through multiple signalling pathways.

Hypoxic ischemia is one of the primary causes of neonatal brain injury [67]. Mitochondrial autophagy plays a crucial role in the degradation of damaged mitochondria and cell survival following neonatal brain hypoxic ischemia (HI) injury [68]. Wu *et al*. reported that the absence of FUNDC1, a novel mitochondrial autophagy receptor, leads to a defect in mitochondrial autophagy, resulting in an imbalance in mitochondrial quality control and metabolic disorders [69]. Under normal circumstances, FUNDC1 interacts with LC3 to form autophagosomes that encapsulate damaged mitochondria, facilitating their fusion and degradation within lysosomes. However, when PHLDA1 is overexpressed, it competes with FUNDC1 for binding to LC3, which is a marker protein for autophagosomes. This competition blocks the formation of autophagosomes and inhibits autophagy. Further research has demonstrated that the knockout of PHLDA1 alleviates the inhibition of FUNDC1, thereby enhancing mitochondrial autophagy and exerting a protective effect on neurons in ischemic and hypoxic neonatal rats [65].

BHLHE40 (e40), a member of the basic helix-loop-helix family, has a protective effect on the pathological processes of neurogenic diseases [70]. During the treatment of ischemic cerebrovascular disease, cerebral ischemia/reperfusion (I/R) injury can result in neuronal cell death and neurological dysfunction [71]. PHLDA1 has been identified as a promoter of neuronal damage in brain I/R injury [64]. Its detrimental effects on neuronal cells are primarily mediated through the inhibition of mitochondrial autophagy, the enhancement of oxidative stress, and the promotion of apoptosis. BHLHE40, a transcriptional suppressor, plays a critical role in the hypoxic response, inflammatory regulation, and cell survival [72]. It exerts a protective effect by recognizing and binding to a specific sequence in the promoter region of the PHLDA1 gene *via* its DNA-binding domain, thereby inhibiting PHLDA1 transcription. In rat models of I/R injury and primary hippocampal neuronal hypoxia/reoxygenation (OGD/R), the transcription factor BHLHE40 can mitigate cell damage by regulating PHLDA1 transcription, ultimately preventing brain I/R injury [73].

Ischemic cerebrovascular disease is frequently accompanied by mitochondrial dysfunction and endoplasmic reticulum (ER) stress. The disruption of these organelles can directly lead to cell death through various signalling pathways [74]. Peroxisome proliferator-activated receptors (PPARs) are a class of nuclear receptors that function as transcription factors, regulating the expression of genes. PPARs regulate the expression of genes involved in metabolic homeostasis and multiorgan function, playing crucial roles in various physiological processes, including lipid metabolism, energy balance, inflammatory responses, and cell differentiation [75]. In neuronal studies, the activation of PPARγ has been demonstrated to have anti-apoptotic effects, protecting neurons from diverse types of damage, thereby positioning PPARγ as a potential therapeutic target for cerebral ischemia/reperfusion therapy [76, 77]. PHLDA1 levels are significantly elevated in middle cerebral artery occlusion/ reperfusion (MCAO/R)-treated mice and in hypoxic-glucose/ reoxygenation (OGD/R)-stimulated neurons, which serve as *in vivo* and *in vitro* models of ischemia/reperfusion, respectively. The knockout of PHLDA1 promotes the nuclear translocation of PPARγ, potentially mediating the reversal of mitochondrial function and alleviation of ER stress [25].

The role of neuroinflammation in ischemic stroke has attracted increasing attention, especially with respect to the transformation of proinflammatory and anti-inflammatory microglia. In the ischemia-reperfusion model, knockdown of PHLDA1 can reduce the expression of proinflammatory markers (such as CD16, TNF-α, IL-6, IFN-γ, and iNOS) while increasing the expression of anti-inflammatory markers (such as CD206, IL-4, IL-10, and Arg-1), thereby promoting the transformation from the proinflammatory phenotype to the anti-inflammatory phenotype. Furthermore, PHLDA1 knockdown can reduce the expression of NLRP3, ASC, caspase-1, and IL-1β, inhibit the activation of the NLRP3 inflammasome, and effectively mitigate brain injury induced by ischemia-reperfusion [78].

Intracerebral hemorrhage (ICH) is a form of brain damage resulting from acute parenchymal hemorrhage due to the rupture of cerebral vessels and represents the deadliest type of acute stroke [79]. Studies have demonstrated a significant association between pyroptosis and the progression of secondary brain injury following intracerebral hemorrhage (ICH). Hematoma components, including hemoglobin and iron ions, activate the TLR4/NF-κB signalling pathway in microglia and macrophages, leading to the upregulation of NLRP3 and pro-IL-1β expression, which subsequently induces pyroptosis in these cells [80]. Additionally, heme released from erythrocyte lysate after ICH can directly activate caspase-4/5/11, splice GSDMD, and induce pyroptosis independently of NLRP3 [81]. Inhibiting pyroptosis is anticipated to be a therapeutic strategy aimed at mitigating brain injury after ICH [82]. Early growth response factor 1 (Egr1) is a transcription factor involved in tissue injury, immune responses, and the formation of fibrous tissue. Recent findings suggest that Egr1 may be linked to neuronal pyroptosis [83]. Following intracerebral hemorrhage (ICH), components of the hematoma, such as hemoglobin and thrombin, induce the expression of Egr1 *via* the MAPK/ERK signalling pathway. Egr1 subsequently binds to the GC-rich sequence located in the promoter region of PHLDA1 (*e.g*., 5'-GCGGGGGCG-3'), thereby recruiting p300/CBP to increase PHLDA1 transcription. Wang *et al*. demonstrated that Egr1 can upregulate PHLDA1. Concurrently, PHLDA1 elevates oxidative stress through the Rac1/NOX2 pathway, which triggers Nlrc4 oligomerization. Activated Nlrc4 then recruits ASC and procaspase-1, leading to the formation of inflammasome complexes that promote neuronal pyroptosis [84].

Subarachnoid hemorrhage (SAH) is a catastrophic acute cerebrovascular event characterized by a poor prognosis and a high incidence of neurocognitive dysfunction and is closely related to early brain injury (EBI) induced by SAH [85]. Neuroinflammation has been identified as a critical factor in the progression of EBI following SAH [86, 87]. Following subarachnoid hemorrhage (SAH), the differentiation of microglia into proinflammatory and anti-inflammatory phenotypes plays a crucial role in determining neuronal injury and repair [88]. Excessive activation of the NLRP3 inflammasome can drive proinflammatory polarization toward microglia, thereby exacerbating inflammatory damage. Studies have shown that knocking down PHLDA1 may promote the transformation of microglia from a proinflammatory phenotype to an anti-inflammatory phenotype by inhibiting the activation of NLRP3, which in turn improves brain injury induced by SAH [40].

### 4.2 PHLDA1 and its Relationship with Epilepsy Warrant Further Exploration

Epilepsy is a prevalent neurological disorder affecting more than 70 million individuals worldwide. It is particularly common among children, adolescents, and elderly individuals, with approximately 30% of cases classified as refractory epilepsy. This condition ranks as the second most common neurological disorder, following cerebrovascular diseases [89]. Hippocampal sclerosis is a significant etiology of epilepsy and is characterized by specific patterns of neuronal loss and gliosis [90]. In the sclerotic hippocampal tissue of patients with refractory epilepsy, PHLDA1 is markedly upregulated in both viable neurons and reactive astrocytes [91]. Neuronal loss and gliosis, which accompany the onset and progression of epilepsy, are distinguishing features of this disorder and are especially pronounced in patients experiencing recurrent or acute episodes [92]. During the progression of epilepsy, the central nervous system, particularly vulnerable regions such as the hippocampus, exhibits abnormal expression of numerous apoptosis-related proteins (*e.g*., Bax, caspase-3, and Fas). These proteins ultimately lead to programmed neuronal death *via* the activation of either the mitochondrial apoptotic pathway or the death receptor pathway [93]. Research has shown that the expression level of PHLDA1 is significantly increased in the pathological brain tissues of epilepsy patients and animal models. This upregulation may increase Bax/BAK-mediated mitochondrial apoptosis by inhibiting Akt signalling, promoting Fas-dependent extrinsic apoptosis, or inhibiting the Nrf2 antioxidant pathway, thereby exacerbating oxidative stress and leading to neuronal loss [94]. Concurrently, glial activation represents a principal pathological feature of hippocampal sclerosis. Studies have demonstrated that glial activation can drive the expression of PHLDA1, with activated glial cells secreting IL-1β and TNF-α, which induce PHLDA1 transcription through the NF-κB or JAK-STAT signalling pathways [17, 35].

In contrast to previous studies reporting that the IGFR/p38 MAPK pathway activates IGF-1, which in turn upregulates PHLDA1 to protect against cellular damage, IGF-1 levels are notably low in epileptic conditions. The role of the p38 pathway in epilepsy is complex and multifaceted [95], and it is closely linked to the glial activation of inflammatory responses [96]. Although relevant data were not provided in this study, these findings suggest that PHLDA1 is closely associated with neuronal apoptosis and the abnormal proliferation of glial cells, which are among the pathogenic characteristics of epilepsy.

### 4.3 PHLDA1 is Associated with Amyotrophic Lateral Sclerosis (ALS)

Amyotrophic lateral sclerosis (ALS) is a progressive and fatal disease characterized primarily by neuronal degeneration and necrosis [97]. PHLDA1 has been identified as a key regulator of neuronal death through its involvement in apoptosis, the immune response, oxidative stress, and various other pathways. Furthermore, mutations in Cu/Zn superoxide dismutase (SOD1) are closely associated with the onset of ALS [98]. In experimental mouse models with SOD1 mutations, heat shock proteins (HSPs) and PHLDA1 reportedly disturb the balance between cell survival and apoptosis, a critical factor in cell death in the ALS model [99]. HSF-1, recognized as a transcription factor of heat shock proteins (HSPs), has also been confirmed as a transcriptional regulatory factor for PHLDA1. Specifically, HSF-1 binds to the heat shock response element located in the promoter region of the PHLDA1 gene, resulting in chromatin contraction and the inhibition of PHLDA1 transcription. This process effectively suppresses the proapoptotic effects associated with PHLDA1 [100]. However, with aging, the levels of HSPs in neurons and glial cells in model mice decrease, and the progressive reduction in HSF1 expression leads to a reversal of the balance between HSPs and PHLDA1. Consequently, the expression of PHLDA1 in glial cells increases, whereas its expression in neurons progressively decreases [101]. The disturbance of homeostasis among HSPs, HSF-1, and PHLDA1, along with gliosis and PHLDA1 involvement in the Fas pathway [94], is closely related to the progressive loss of neurons.

### 4.4 PHLDA1 is Associated with PD

PD is a chronic condition characterized primarily by movement disorders and the progressive degeneration of dopaminergic neurons in the substantia nigra of the brain [102]. This pathological process involves oxidative stress, mitochondrial dysfunction, neuroinflammation, and abnormal protein aggregation. PHLDA1 may be implicated through several mechanisms. Specifically, PHLDA1 reduces the expression of antioxidant enzymes by mediating the GSK-3β-induced degradation of Nrf2, thereby increasing the vulnerability of neurons to reactive oxygen species (ROS) attacks [47, 64]. Microglia-mediated neuroinflammation plays a crucial role in the progression of neurodegenerative diseases, including PD [103]. PHLDA1 plays a crucial role in immune regulation, particularly in toll-like receptor-mediated immune responses. In MPTP-induced mouse models of PD, PHLDA1 expression is markedly increased. The overexpression of PHLDA1 leads to a direct interaction with TRAF6, enhancing K63-linked ubiquitin-mediated NF-κB signalling activation, which promotes the production of proinflammatory factors such as TNF-α, IL-1β, iNOS, and COX-2, thereby exacerbating neuronal damage [35]. Although the study of PHLDA1 in PD is still in its nascent stages, its involvement in apoptosis, oxidative stress, and the inflammatory response suggests that it may play a pivotal role in the pathogenesis of PD. Future research is anticipated to elucidate its specific mechanisms and provide novel insights into the treatment of PD.

PHLDA1 plays a significant role in the pathogenesis of neurological diseases. A summary is shown in Fig. (**5**). Other members of the PHLDA family, namely, PHLDA2 and PHLDA3, also exhibit numerous connections to the pathogenesis of these diseases. This article provides a concise summary, as illustrated in Table **1**, with the intention of offering insights for the diagnosis and treatment of these conditions.

## 5 INTERVENTION STRATEGIES TARGETING PHLDA1

Intervention with PHLDA1 is approached from the perspective of molecular targeting and regulatory mechanisms, primarily through the modulation of signalling pathways. With respect to the JAK2-ERK1/2-STAT3 pathway, TLR2 agonists such as Pam3CSK4 can upregulate PHLDA1 *via* this pathway, thereby promoting inflammatory responses [17]. The JAK2 inhibitor AG490 can inhibit PHLDA1 expression, alleviating neuroinflammation and microglial cell activation in models of Parkinson's disease [35]. In the p53-dependent pathway, PHLDA1, as a target gene of p53, can induce apoptosis in glioma by regulating DNA damage, with its expression being modulated by the SNHG1/miR-194 axis [110]. Genetic technology is currently utilized primarily for constructing PHLDA1 gene knockout cell models. Adeno-associated virus (AAV)-mediated silencing of PHLDA1 has demonstrated therapeutic potential in neurological diseases, although challenges related to tissue targeting and long-term safety still need to be addressed. The research and development of pharmacological regulators remain in the experimental phase. Among these, small molecule inhibitors and activators, in addition to the JAK2/STAT3 inhibitor AG490, include the Aurora A kinase inhibitor MK-5108, which can combine with anti-GD2 antibodies to increase neuroblastoma cell death by reducing PHLDA1 levels [111]. In an emphysema model, the Nrf2 activator TBHQ decreases alveolar epithelial apoptosis by inhibiting Stub1-mediated degradation of PHLDA1 [112]. Research on natural compounds and their derivatives is relatively limited. Silybin nanoformulations have been found to induce apoptosis in hepatoma cells through the miR-155-3p/SOCS2/PHLDA1 axis [26-29]. Moreover, miR-194 has emerged as a promising anti-inflammatory molecule that improves hepatic ischemia/ reperfusion injury by targeting PHLDA1 in a TRAF6-dependent manner [42]. PHLDA1 is regulated primarily in neurological diseases through the application of small-molecule inhibitors, agonists, and gene knockout techniques. Furthermore, the majority of these interventions are currently in the preclinical stage, necessitating the advancement of large-scale animal experiments and toxicity evaluations. The summary is shown in Table **2**.

## CONCLUSIONS AND PROSPECTS

Currently, most research on PHLDA1 has focused on its role in tumors. As investigations progress, the structure and biological characteristics of PHLDA1 are being increasingly elucidated and confirmed. This article primarily discusses the typical and atypical biological effects of PHLDA1, along with the associated signalling pathways, emphasizing its crucial role in neurosystem-related diseases and thereby enhancing the understanding of PHLDA1. Moreover, the function of PHLDA1 varies across different diseases and cell types and is linked to its oligomerization, subcellular localization, and posttranslational modifications. Various signalling pathways intricately regulate the biological activity of PHLDA1, allowing cells to adapt to diverse physiological conditions. Consequently, PHLDA1 may have a potential connection with neurological diseases, positioning it as a promising research avenue for therapeutic strategies and establishing it as a potential new target for clinical interventions in neurological disorders. Although existing studies have revealed the significance of PHLDA1 in nerve injury, many aspects regarding its specific role and mechanism are still unknown. First, some studies suggest that PHLDA1 promotes neuronal apoptosis and neuroinflammation and is a neurotoxic factor. However, other studies have indicated that the absence of PHLDA1 can exacerbate oxidative stress-induced apoptosis, which may be related to the type, intensity, and duration of stress. Second, the roles of PHLDA1 in neurons, astrocytes, microglia, and oligodendrocytes may be completely different. Most of the existing studies have been conducted in mixed cell systems and are unable to distinguish their cell-specific functions. Moreover, their distributions in different organelles may also have different functions. Third, at different stages of the disease, such as the acute, subacute, and recovery stages, PHLDA1 may play different roles, and its specific functions still need to be further studied and clarified. Fourth, PHLDA1 is considered an "adaptor protein", and its direct interaction proteome in the nervous system has rarely been explored. Currently, how it "connects" upstream signals to complex downstream pathways remains unknown. The fifth point is that, currently, almost all research is focused on acute injury models (stroke, ischemia, hemorrhage). However, the role of PHLDA1 in chronic neurodegenerative diseases (such as Alzheimer's disease, multiple sclerosis, and Huntington's disease) and neuropsychiatric disorders (such as depression) has rarely been studied. In conclusion, PHLDA1 is a molecule with complex functions and great potential, but has not yet been extensively studied in neurological diseases. Future research needs to shift from "whether it is important" to more refined questions, such as "how it is important", "in which cells it is important", and "when it is important", ultimately providing new targets and ideas for the treatment of neurological diseases.

## Figures and Tables

**Fig. (1):**
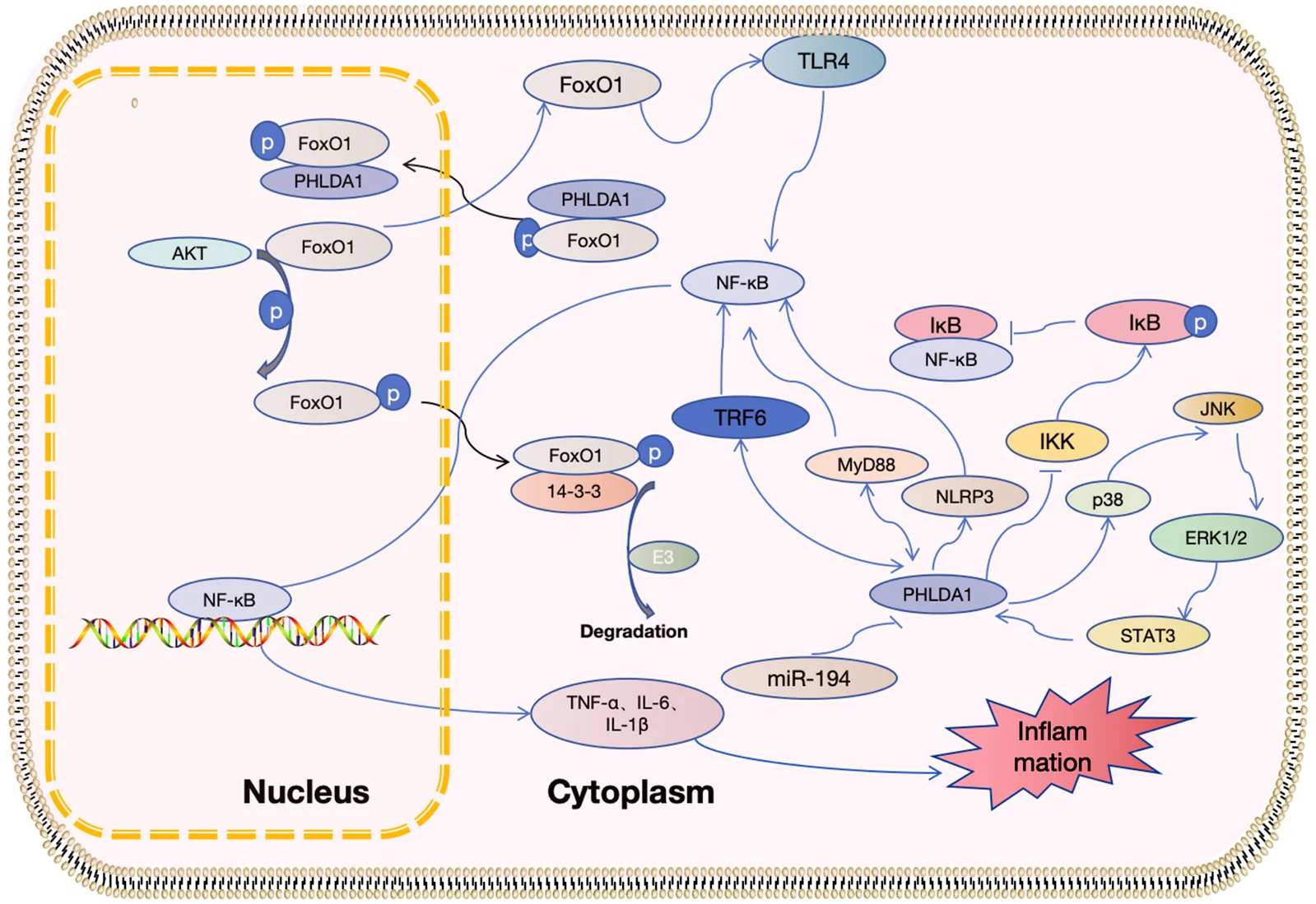
Inflammatory signalling pathways regulated by PHLDA1. Forkhead box protein O1 (FoxO1), a member of the FoxO family, is predominantly localized in the nucleus and functions by regulating the transcription of specific genes. It acts as a transcriptional regulator of toll-like receptor (TLR4) and its proinflammatory pathways, directly activating the TLR4 gene and downstream nuclear factor kappa-B (NF-κB) and mitogen-activated protein kinase (MAPK) signalling pathways, which enhance the production of proinflammatory cytokines such as tumor necrosis factor-α (TNF-α), interleukin-1 beta (IL-1β), and interleukin-6 (IL-6), thereby promoting inflammation. The protein kinase B (AKT) pathway is the primary route for FoxO1 phosphorylation. Once phosphorylated, FoxO1 translocates from the nucleus to the cytoplasm, where it binds to 14-3-3 chaperone proteins and subsequently associates with ubiquitin E3 ligases for degradation. PHLDA1 can competitively bind to FoxO1, displacing 14-3-3 proteins and retaining FoxO1 in the nucleus, thereby exerting its function. Additionally, PHLDA1 interacts with the inhibitor of κB kinase (IKK) complex, inhibiting IKK activity and promoting the phosphorylation and degradation of inhibitor of κB (IκB), which facilitates the nuclear translocation and transcriptional activity of NF-κB. PHLDA1 can enhance NF-κB function through TNF receptor-associated factor 6 (TRAF6), myeloid differentiation primary response gene 88 (MyD88), and NLR family pyrin domain containing 3 (NLRP3). Furthermore, PHLDA1 regulates the JUN N-terminal kinase-extracellularly regulated protein kinase 1/2-signal transducer and activator of transcription 3 (JNK-ERK1/2-STAT3) signalling pathway *via* p38 mitogen-activated protein kinase (p38), while STAT3 can also promote PHLDA1 expression. Notably, miR-194 can inhibit PHLDA1 expression.

**Fig. (2):**
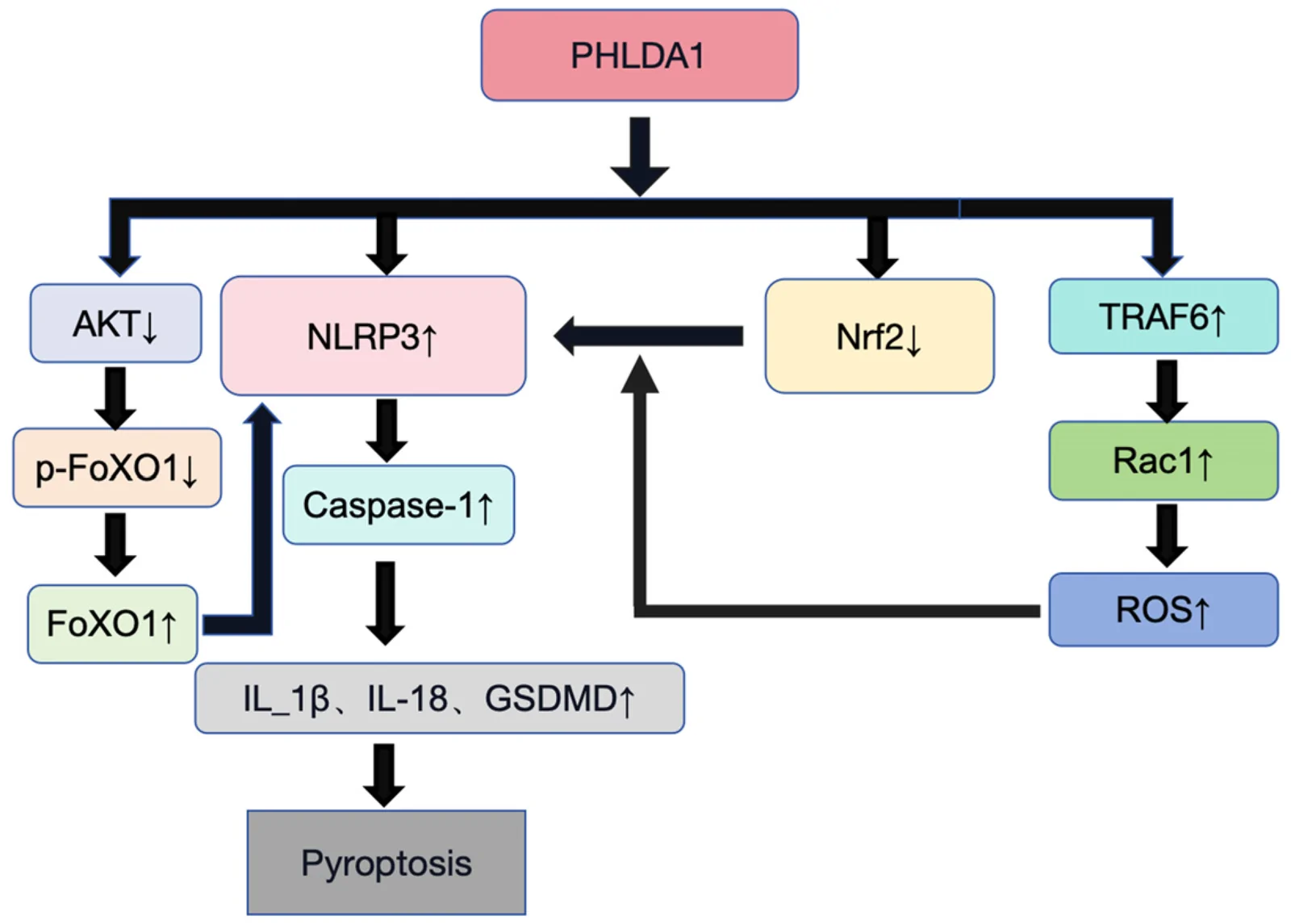
Pyroptosis signalling pathways regulated by PHLDA1. Akt promotes the phosphorylation of forkhead box protein O1 (FOXO1), which results in the downregulation of pyroptosis-related proteins, including NLR family pyrin domain containing 3 (NLRP3) and interleukin-1 beta (IL-1β), and inhibits the activation of gasdermin D (GSDMD). Additionally, PHLDA1 can inhibit protein kinase B (AKT) by competitively binding to phosphatidylinositol (PIP), thereby promoting pyroptosis. PHLDA1 directly activates NLRP3, leading to caspase 1-mediated increases in IL-1β and interleukin-18 (IL-18), which induce pyroptosis. Furthermore, PHLDA1 suppressed the expression of nuclear factor erythroid 2-related factor 2 (Nrf2), and the subsequent inhibition of Nrf2 activated NLRP3. PHLDA1 also enhances the expression of TNF receptor-associated factor 6 (TRAF6), which activates Ras-related C3 botulinum toxin substrate 1 (Rac1). Activated Rac1 promotes the generation of reactive oxygen species (ROS) through nicotinamide adenine nucleotide phosphate (NADPH) oxidase, thereby facilitating the assembly and activation of NLRP3.

**Fig. (3):**
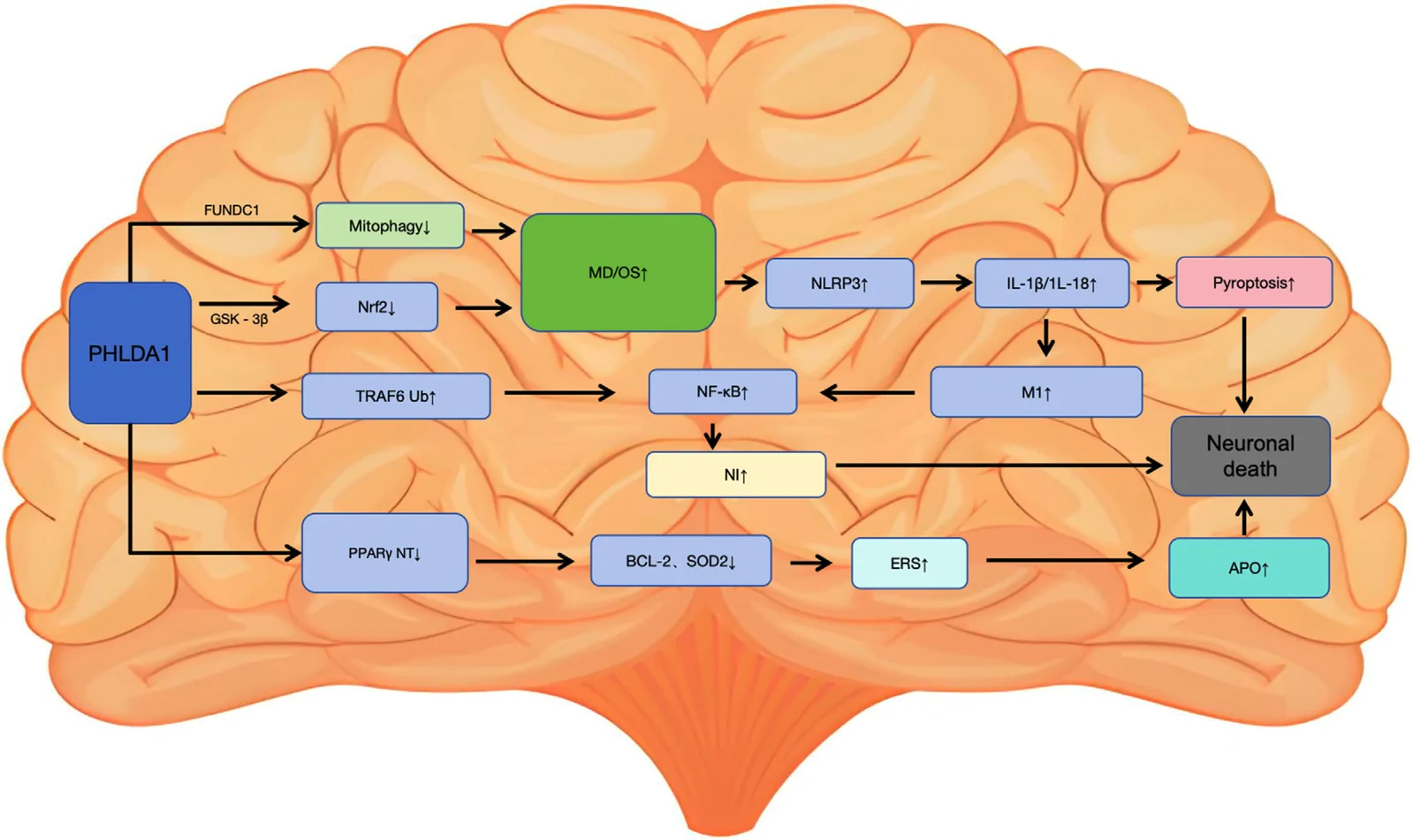
Schematic diagram of the role of PHLDA1 in the interaction of neural pathways. PHLDA1 induces mitochondrial autophagy (MD) and oxidative stress (OS) by regulating FUN14 domain 1 (FUNDC1) and nuclear factor erythroid 2-related factor 2 (Nrf2), thereby activating the NLR family pyrin domain 3 (NLRP3). It leads to the activation of interleukin-1β (IL-1β) and interleukin-18 (IL-18), ultimately resulting in pyroptosis. Additionally, the activation of IL-1β and IL-18 enhances nuclear factor kappa-B (NF-κB) activity, exacerbating neuroinflammation (NI) and ultimately contributing to neuronal death. Furthermore, PHLDA1 promotes the ubiquitination of TNF receptor-associated factor 6 (TRAF6 Ub), which activates NF-κB and further stimulates neuroinflammation. Upon interaction with peroxisome proliferator-activated receptor gamma (PPARγ), PHLDA1 inhibits its nuclear translocation, resulting in a decrease in the expression of antipyroptotic genes, including B-cell lymphoma-2 (BCL-2) and Cu/Zn-superoxide dismutase 2 (SOD2). This interaction exacerbates endoplasmic reticulum stress (ERS), ultimately leading to cell death *via* apoptosis (APO).

**Fig. (4):**
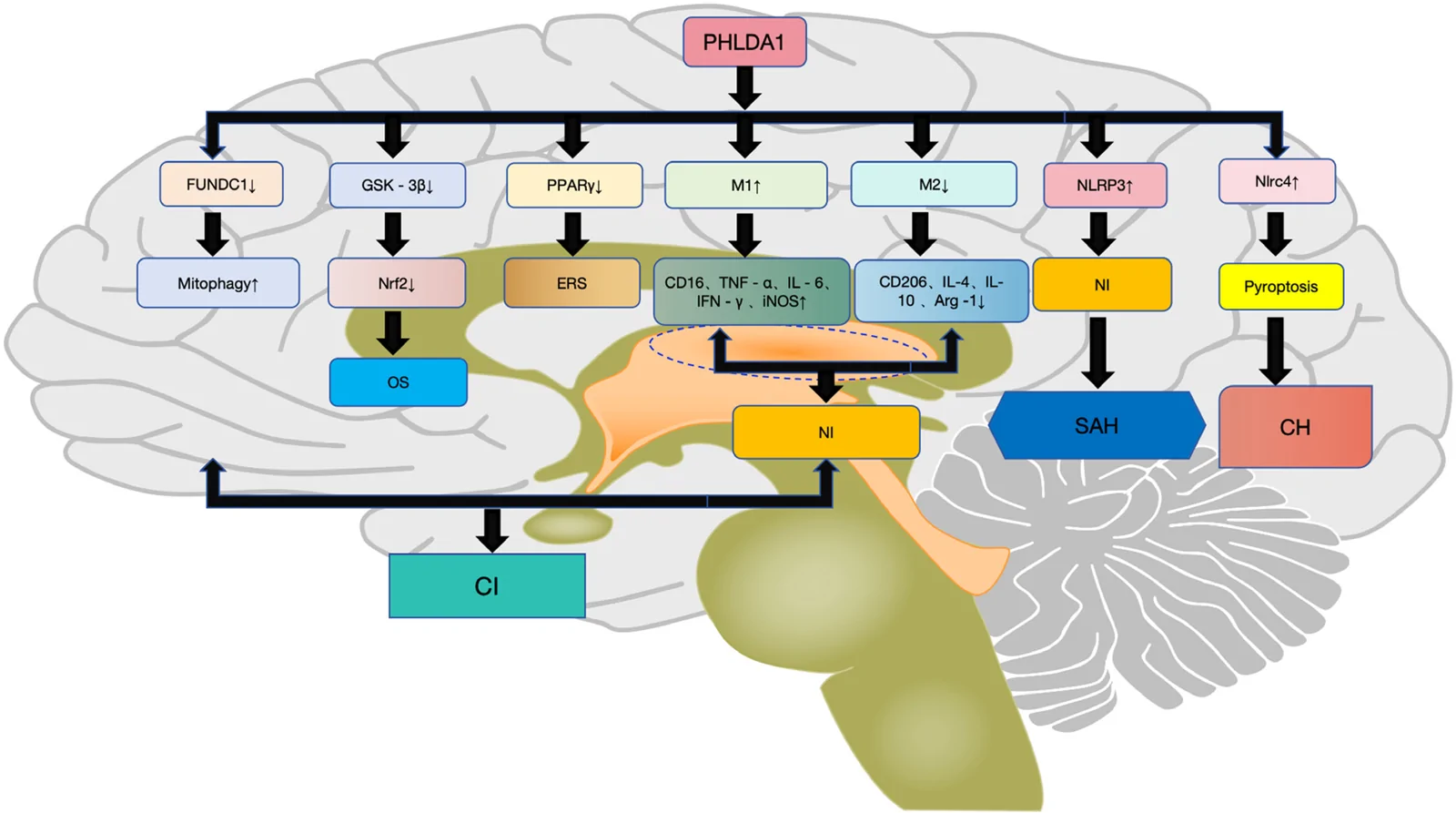
Brain vascular-related signalling pathways regulated by PHLDA1. PHLDA1 inhibits the expression of FUN14 domain-containing 1 (FUNDC1), thereby enhancing mitochondrial autophagy. Additionally, PHLDA1 mediates oxidative stress (OS) by inhibiting glycogen synthase kinase 3β (GSK-3β) and weakening the signalling of nuclear factor erythroid 2-related factor 2 (Nrf2). It also inhibits the nuclear translocation of peroxisome proliferator-activated receptor gamma (PPARγ), which mediates mitochondrial dysfunction and exacerbates endoplasmic reticulum stress (ERS). Furthermore, PHLDA1 increases the expression of proinflammatory microglial markers, such as cluster of differentiation 16 (CD16), tumor necrosis factor-α (TNF-α), interleukin-6 (IL-6), interferon-gamma (IFN-γ), and inducible nitric oxide synthase (iNOS), while reducing the expression of anti-inflammatory microglial markers, such as cluster of differentiation 206 (CD206), interleukin-4 (IL-4), interleukin-10 (IL-10), and arginase-1 (Arg-1). This shift converts the proinflammatory phenotype to the anti-inflammatory phenotype, thereby inducing neuroinflammation (NI). These pathways are relevant to the cerebral infarction (CI) model. In a subarachnoid hemorrhage (SAH) model, PHLDA1 activated NLR family pyrin domain-containing 3 (NLRP3), promoting neuroinflammation. In the intracerebral hemorrhage (IH) model, PHLDA1 mediates pyroptosis by promoting the expression of the nucleotide-binding oligomerization domain, leucine-rich repeat, and caspase recruitment domain-containing 4 (NLRC4).

**Fig. (5):**
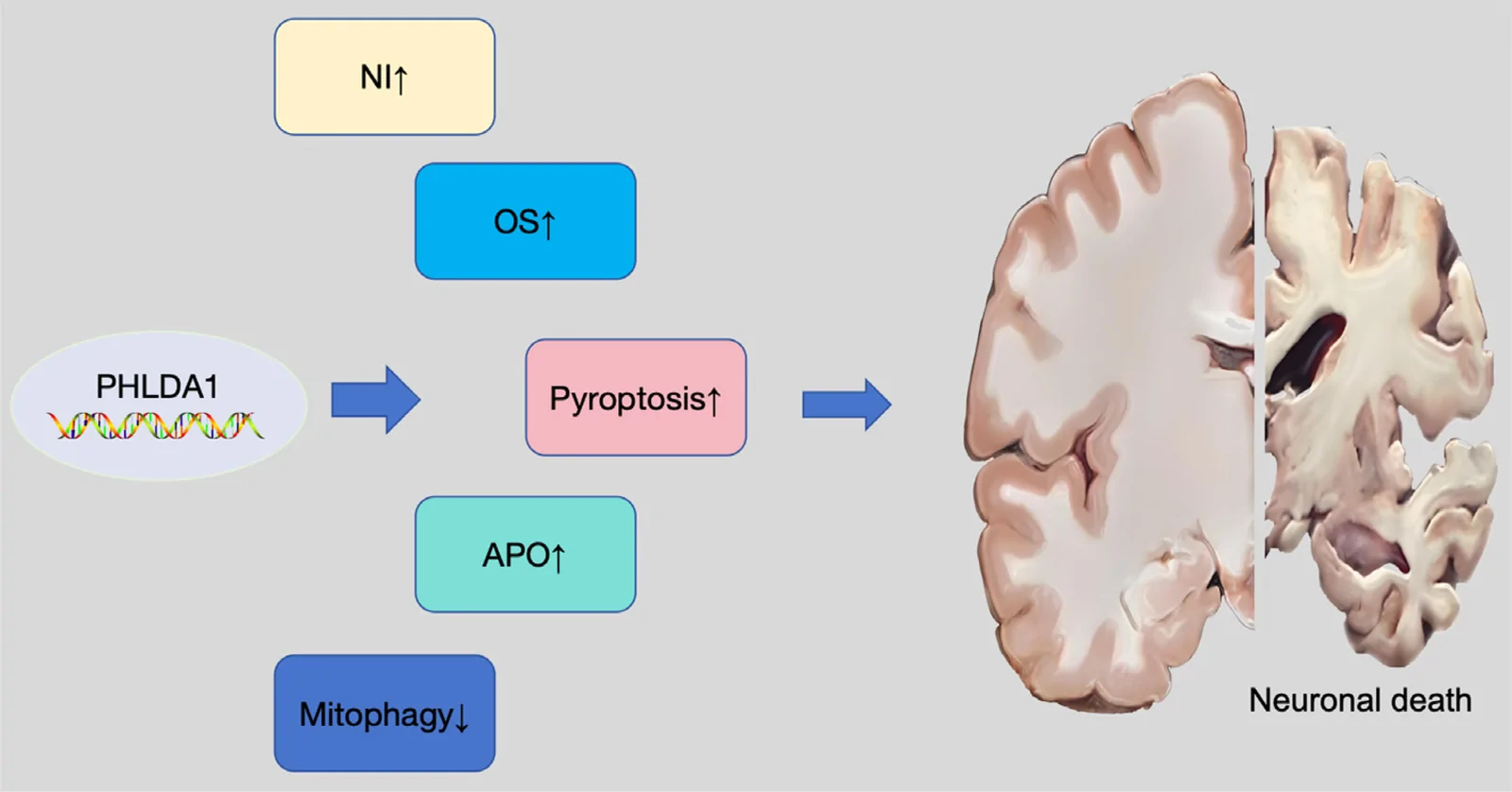
Summary diagram of the action of PHLDA1. PHLDA1 mediates the death of nerve cells through multiple mechanisms, mainly by promoting neuroinflammation (NI), oxidative stress (OS), pyroptosis, and apoptosis (APO), and inhibiting mitochondrial autophagy (mitophagy).

**Table 1:** The underlying mechanisms of PHLDA family members in neurological diseases.

**Molecule**	**Disease/Model**	**Mechanism of Action**	**Potential Clinical Applications**
PHLDA1	Hypoxic-ischemic brain injury	Aggravating brain injury by inhibiting FUNdC1-mediated mitochondrial autophagy [66-69]	Hypoxia-ischemia, stroke, SAH and ICH models all showed that their levels were positively correlated with infarction/edema and neurological scores, and have been proposed as ultraearly serum markers to predict 24-hour edema and cognitive outcomes. Downregulation of siRNA/CRISPR or HF-1 activators can reduce infarction and improve behavior. The animal efficacy has been completed, and preclinical applications for perinatal brain protection, intravascular postoperative anti-inflammation, and HER2 resistance reversal are being advanced.
	Cerebral ischemia-reperfusion injury	Alleviate neuronal apoptosis and mitochondrial dysfunction through the PPARγ pathway [23-25]	
	Cerebral hemorrhage model	Egr1 promotes NLRC4-dependent neuronal pyroptosis by upregulating PHLDA1 [74-84]	
	Subarachnoid hemorrhage	Regulate microglial responses and the NLRP3 inflammasome signalling pathway [34-40]	
	Parkinson's disease	Regulate microglia-mediated neuroinflammation through K63 ubiquitination of TRAF6 [31-35]	
	Epilepsy	IGFR/p38 MAPK activates IGF-1, upregulates PHLDA1 and induces neuronal apoptosis [77-95]	
	ALS	The progressive reduction in HSF1 expression leads to the reversal of the balance between HSPs and PHLDA1, promoting neuronal degeneration and death [80-99]	
PHLDA2	Glioma	Knockdown of PHLDA2 promotes apoptosis and autophagy through the AKT/mTOR pathway [104]	The high expression of PHLDA2 in glioma inhibits apoptosis and autophagy through the AKT/mTOR pathway, leading to a poor prognosis for patients. Knocking down PHLDA2 can enhance the efficacy of temozolomide and prolong survival, making it a potential therapeutic target. On the other hand, hypomethylation and elevated expression of placental PHLDA2 are associated with fetal growth restriction and neurodevelopmental risks. Currently, they have been included in the multigene detection panel for early warning of brain development in premature infants, and the safety of amniotic cavity administration of the demethylating drug 5-aza-dC is being evaluated in animal models.
	Neural development	Abnormal expression of PHLDA2 in the placenta is associated with fetal growth restriction and neurobehavioral abnormalities [105]	
	Cognitive and behavioral disorders	Insufficient endocrine function of the placenta leads to abnormal expression of imprinted genes such as PHLDA2, which affects the cognitive and social behaviors of the offspring [106]	
PHLDA3	Cerebral ischemia-reperfusion injury	Reduce oxidative stress and inflammatory responses through the PI3K/AKT/Nrf2 pathway [107]	In cerebral ischemia-reperfusion and ALS models, the AAV vector overexpressing PHLDA3 alleviates oxidative stress and prolongs the survival of motor neurons by activating the PI3K-AKT-Nrf2 pathway, thereby extending the survival period of animals by approximately 11%. Its GMP-grade AAV9 formulation has completed toxicological studies and is preparing to submit a Pre-IND application, becoming the only candidate gene among the three to have completed the drugability validation of the therapeutic vector.
	Neuroendocrine tumor	The p53-PHLDA3-Akt axis regulates tumorigenesis (such as pancreatic neuroendocrine tumors) [108]	
	ALS	PHLDA3 can promote neurotoxicity in SOD1-mutated astrocytes [109]	

**Table 2:** Comparison table of PHLDA1 regulators.

**Type of Regulator**	**Representing Molecules/Compounds**	**Mechanism**	**Research and Development Status**
Hypoxic-ischemicSmall molecule preparations	Pam3CSK4	TLR2 agonist. Upregulate PHLDA1 through the JAK2-STAT3 pathway.	Preclinical research
	AG490	Inhibit the JAK2-STAT3 pathway and reduce the expression of PHLDA1.	Preclinical research
	MK-5108	MK-5108 can be combined with anti-GD2 antibodies to reduce the level of PHLDA1.	Preclinical research
	TBHQ	TBHQ mediates the degradation of PHLDA1 by inhibiting STUB1	Preclinical research
Gene editing therapy	shRNA-PHLDA1	Silence PHLDA1 expression	Verification of animal models
Derivatives of natural compounds	Silybin nanoformulations	Increase the expression of PHLDA1 through the miR-155-3p/SOCS2 axis.	Preclinical research

## LIST OF ABBREVIATIONS

AG490: Tyrphostin AG490
AKI: Acute Kidney Injury
AKT: Protein Kinase B
ALI: Acute Lung Injury
ALS: Amyotrophic Lateral Sclerosis
Arg-1: Arginase-1
ASC: Apoptosis-associated Speck-like Protein Containing
Bak: BCL-2 Homologous Antagonist/Killer
Bax: BCL-2-associated X Protein
Bcl-2: B-cell Lymphoma-2
BH-3: BCL-2 Homology Domain 3
BHLHE40: Basic Spiral-ring-spiral Family Member e40
Caspase-1: Cysteine-requiring Aspartate Protease-1
CBP: CREB-binding Protein
CD16: Cluster of Differentiation 16
CD206: Cluster of Differentiation 206
COX-2: Cyclooxygenase-2
CRM1: Chromosome Region Maintenance 1
Cyt c: Cytochrome c
DIABLO: Direct IAP-Binding Protein with Low PI
EBI: Early Brain Injury
Egr1: Early Growth Response Factor 1
ER: Endoplasmic Reticulum
ERK: Extracellular Regulated Protein Kinases
FGF21: Fibroblast Growth Factor 21
FoxO1: Forkhead Box Protein O1
FUNDC1: FUN14 Domain Containing 1
GCLC: Glutamate-cysteine Ligases
GSDMD: Gasdermin D
GSK-3β: Glycogen Synthase Kinase-3β
HCC: Hepatocellular Carcinoma
HI: Hypoxic Ischemia
HO-1: Haem Oxygenase 1
HSF-1: Heat Shock Factor 1
HSPs: Heat Shock Proteins
I/R: Ischemia/Reperfusion
ICH: Intracerebral Hemorrhage
IFN-γ: Interferon-gamma
IGF1: Insulin-like Growth Factor 1
IKB: Inhibitor of κB
IKK: Inhibitor of κB Kinase
IL-10: Interleukin-10
IL-18: Interleukin-18
IL-1β: Interleukin-1 beta
IL-4: Interleukin-4
IL-6: Interleukin-6
iNOS: Inducible Nitric Oxide Synthase
IRE1: Inositol-requiring Enzyme 1
JAK2: Janus kinase-2
JNK: JUN N-terminal Kinases
LC3: Microtubule-Associated Protein 1 Light Chain 3
LPS: Lipopolysaccharide
MAPK: Mitogen-Activated Protein Kinase
MCAO/R: Middle Cerebral Artery Occlusion/Reperfusion
MEF: Embryonic Fibroblasts
MK-5108: MK-5108 Hydrochloride
MOMP: Major Outer Membrane Protein
MPTP: Mitochondrial Permeability Transition Pore
mTOR: Mechanistic TARGET Of rapamycin
MyD88: Myeloid Differentiation Primary Response Gene 88
NADPH: Nicotinamide Adenine Nucleotide Phosphate
NF-κB: Nuclear Factor Kappa-B
Nlrc4: Nucleotide-binding oligomerization domain, leucine-rich repeat, and caspase recruitment domain-containing 4
NLRP3: NLR Family Pyrin Domain Containing 3
NOX2: NADPH oxidase 2
NQO1: NAD(P)H (quinone redox enzyme 1)
Nrf2: Nuclear Factor Erythroid 2-Related Factor 2
OGD/R: Oxygen-Glucose Deprivation/Reperfusion
p38: p38 Mitogen-activated Protein Kinase
p53: Tumor Protein 53
PD: Parkinsonism
PHLDA1: Pleckstrin Homology Like Domain Family A Member 1
PI3K: Phosphatidyl Inositol 3-kinase
PKC: Protein kinase C
PPARγ: Peroxisome Proliferator-activated Receptor Gamma
Rac1: Ras-related C3 Botulinum Toxin Substrate
ROS: Reactive Oxygen Species
RXRα: Retinoid X Receptor Alpha
SAH: Subarachnoid Hemorrhage
Smac: Second mitochondria-derived activator of caspases
SNHG1: Small Nucleolar RNA Host Gene 1
SOCS2: Suppressor of Cytokine Signalling 2
SOD1: Cu/Zn - Superoxide Dismutase 1
SREBP: Sterol Regulatory Element Binding Protein
STAT3: Signal Transducer and Activator of Transcription 3
STUB1: STIP1 Homologs and U Box-containing Protein 1
TBHQ: Tert - Butylhydroquinone
TDAG51: T-cell Death-related Genes 51
TGF-β: Transforming Growth Factor-β
TLR: Toll-like Receptor
TNF-α: Tumor Necrosis Factor-α
TNFR1: Tumor Necrosis Factor Receptor Type 1
TRAF6: TNF Receptor-Associated Factor 6
XBP1: X-box Binding Protein 1
